# Updates in Air Pollution: Current Research and Future Challenges

**DOI:** 10.5334/aogh.4363

**Published:** 2024-02-01

**Authors:** Dwan Vilcins, Rebecca C. Christofferson, Jin-Ho Yoon, Siti Nurshahida Nazli, Peter D. Sly, Stephania A. Cormier, Guofeng Shen

**Affiliations:** 1 The University of Queensland, Child Health Research Centre, South Brisbane, QLD, Australia; 2 Louisiana State University, School of Veterinary Medicine, Department of Pathobiological Sciences, Baton Rouge, Louisiana, USA; 3 School of Earth Sciences and Environmental Engineering, Gwangju Institute of Science and Technology, Gwangju, 61005, Korea; 4 Faculty of Health Sciences, Universiti Teknologi MARA Cawangan Pulau Pinang, Kampus Bertam, 13200 Kepala Batas, Pulau Pinang, Malaysia; 5 Department of Biological Sciences, Louisiana State University A&M College and the Pennington Biomedical Research Institute, Baton Rouge, Louisiana, 70803, USA; 6 College of Urban and Environmental Sciences, Peking University, Beijing 100871, China

**Keywords:** air pollution, indoor air pollution, climate change, children’s environmental health

## Abstract

**Background:**

The United Nations has declared that humans have a right to clean air. Despite this, many deaths and disability-adjusted life years are attributed to air pollution exposure each year. We face both challenges to air quality and opportunities to improve, but several areas need to be addressed with urgency.

**Objective:**

This paper summarises the recent research presented at the Pacific Basin Consortium for Environment and Health Symposium and focuses on three key areas of air pollution that are important to human health and require more research.

**Findings and conclusion:**

Indoor spaces are commonly places of exposure to poor air quality and are difficult to monitor and regulate. Global climate change risks worsening air quality in a bi-directional fashion. The rising use of electric vehicles may offer opportunities to improve air quality, but it also presents new challenges. Government policies and initiatives could lead to improved air and environmental justice. Several populations, such as older people and children, face increased harm from air pollution and should become priority groups for action.

## Introduction

In 2022, the United Nations declared that every human has the right to a clean environment, including the right to clean air [1]. This landmark resolution highlights how our lives and health are inextricably linked to our environments, which have the potential to promote health or cause harm. The most contemporary example of this link between humans and their environments is the death of Ella Adoo-Kissi-Debrah, aged 9 years, from asthma triggered by air pollution in London in 2013 [2]. The coroner’s report into Ella’s death took the unprecedented action of listing air pollution as an official cause of death and suggested actions be taken to reduce air pollution to prevent avoidable deaths [3]. Ella’s legacy led to a private member’s bill, The Clean Air (Human Rights) Bill, being considered by the UK parliament. Ella’s tragic and preventable death shines a light on the human toll of air pollution, which is commonly presented as statistics: an estimated 6.7 million deaths are caused by air pollution each year [4], 213 million disability-adjusted life years are attributed to air pollution exposure [4], or that air pollution is the fourth highest risk to health globally [5]. This paper will focus on three key areas of air pollution that are important to human health and require more research and that were highlighted during the 2023 Pacific Basin Consortium for Environment and Health conference.

## Indoor air pollution

Indoor air pollution (IAP) is a complex area of environmental health. Our indoor air is governed by many factors, some fixed and some variable, such as regional development status, household socioeconomic status, internal combustion or non-combustion sources, ambient pollution sources, the external environment, the design and construction of the building, and the occupants and activities occurring within that space [6,7]. The amount and composition of IAP are defined by these factors. In addition to the criteria air pollutants of concern, such as particulate matter and inorganic gaseous pollutants (CO, NO_2_), indoor spaces can contain high levels of biological pollutants and organic gaseous pollutants, such as volatile organic compounds [6]. It has been argued that until we fully understand and account for indoor air pollution in addition to ambient air pollution, we cannot truly understand the effect of air pollution on health [8].

For many populations, the majority of their time is spent indoors: in homes, workplaces, schools, vehicles, and recreation facilities [7]. It has previously been reported that an adult living in the US spends up to 87% of their time indoors [7]. While this fraction may vary in different countries and populations, several national surveys on exposure activities suggest that the global average is likely to be similar [8]. It is not just homes where exposure to air pollution occurs. Children can be exposed to indoor air pollution in schools too, and exceedances above safe limits for certain pollutants have been seen [9,10,11]. A focus on green buildings does not in itself guarantee good indoor air quality (IAQ), with evidence showing that green buildings perform no better or worse than conventional designs with pollutants such as PM_2.5_, ozone, and NO_2_ [12]. However, green buildings with a focus on natural ventilation built into their design can be cheaper to run and maintain and improve IAQ [13].

Lack of access to clean fuels for cooking and heating remains an important driver of IAP in domestic settings in low-income countries and some regions of high-income countries. A large portion of the 3.8 million deaths per year due to IAP is driven by dirty solid fuels being used as an energy source in homes [14]. Importantly, pollutants generated from the use of low-efficiency solid fuels inside homes contribute to the ambient air pollution in these regions [8]. During the COVID-19 pandemic, more solid fuels burned indoors resulted in increased indoor air pollution in low- and middle-income countries (LMICs) and consequent exposure risks, especially for the rural population, despite a reduction in ambient air pollution [15]. This issue continues to require action by the government and non-governmental organisations. The IAQ in high-income countries is also an issue, with cooking, heating, cleaning, burning of combustion materials such as incense, and off-gassing from furniture being major contributors to air pollution in buildings [8]. Modern homes, with their focus on energy efficiency, have become more airtight, which contributes to the buildup of pollutants indoors [6]. In a bid to reduce gaseous air pollutants, the Government of Victoria, Australia, mandated the phase-out of gas heating and cooking, with new homes required to be all-electric by 2024 [16]. However, context matters when it comes to strategies such as these. In areas where biomass burning is the norm, transitioning to gas cooking is a strategy to improve IAQ. The movement of the population can also change IAQ. Countries undergoing rapid urbanisation and migration of rural residents to urban areas for more work opportunities see changes in the household energy structure, leading to distinct combustion-related air pollutant emissions and exposure changes to indoor as well as outdoor air pollution [17].

Indoor air quality is still affected by indoor smoking and/or e-cigarettes in many areas, causing second- and third-hand smoke exposure. The problem of smoking is not resolved, and adequate controls for indoor environments remain an issue. In addition, non-combustion sources produce significant amounts of hazardous components such as VOCs from pharmaceuticals and personal care products and toxic products from indoor chemistry [18]. Some emerging contaminates, like liquid crystal monomers, have been detected in various indoor settings [19], leading to concern about their fates and impacts on human health given the ubiquity of technology in electronic products with liquid crystal displays. Solutions for these emerging contaminants differ from the fuel combustion sources, in which mitigation can focus on clean energy transition and improved ventilation. Controls for other pollutant sources need a systematic synthetic control strategy based on sources, environmental conditions, and behaviour changes.

## Climate change and air pollution

Global climate change will have significant and adverse impacts on air pollution. The interplay between climate and air pollution is both complex and multifactorial, and our ability to model these changes is limited to our understanding of how these relationships play out under current scenarios [20]. Changes in meteorological patterns of temperature, precipitation, and natural disasters will interact with air pollution. For example, more hot and sunny days can lead to the generation of the secondary pollutant ozone [20,21]. Higher temperatures can increase the risk of sulfuric and nitric acid deposition through acceleration of the oxidation process in the atmosphere [20], increase the number and extent of wildfires [22], increase flooding, which may lead to higher exposure to mycotoxins [23], and higher temperatures and CO_2_ will fuel the growth of pollen-producing plants, leading to higher levels of aeroallergens [20]. Increasing temperatures due to global warming could lead to various types of extreme weather and climate events, including drought, heatwaves, and even fire risks.

A recent, dramatic example occurred with the large wildfires in Canada, which have had significant environmental impacts extending far beyond the country’s borders. These fires, often fuelled by dry conditions and sometimes exacerbated by climate change, produce vast amounts of smoke and particulate matter. This smoke can travel thousands of miles, carried by prevailing wind patterns, affecting air quality in distant regions. A notable example is the drift of smoke from Canadian wildfires to New York City, causing a noticeable deterioration in air quality there. The phenomenon highlights the far-reaching consequences of wildfires and the interconnectedness of environmental systems across large geographical distances. In terms of fire weather, it is expected to increase as the global temperature increases [24].

Air pollution due to climate forces from air pollutants and greenhouse gases has a large impact on climate change [25]. Air pollution generally includes a combined exposure of particulate matter (PM) and gases such as CO_2_, CO, O_3_, NOx, and SO_2_ [23]. These pollutants, among others, can both drive climate change and play a role in respiratory and cardiovascular diseases, impairments in the reproductive and central nervous systems, and an increased risk of cancer [26]. Black carbon, methane, tropospheric ozone, and aerosols are among the pollutants that have an impact on the incoming sunlight. These pollutants induce flows that affect other risks from climate change. As an example, climate change as a driver of infectious diseases has also received more widespread attention lately. In particular, warming temperatures in more temperate regions have led and will continue to lead to the expansion of disease-carrying arthropod vectors or the extension of the seasonality of these vectors in areas previously less suitable [27,28,29]. Already, we have realised the consequences of this with the transmission of mosquito-borne viruses in more northern North America, Europe, Argentina, and other temperate ecozones [30,31]. Temperature is a known modifier of viral replication within exothermic vectors, which leads to changes in transmission efficiency and ultimately the probability of (re-)emergence and/or the size of an outbreak [32]. Further, temperature affects the life traits and behaviours of vectors, which can increase the risk of contact between susceptible humans and disease-carrying arthropods [32]. As air pollution and other human activities increase the rate of climate change, we will continue to see these consequences on our risk from vector-borne and other zoonotic diseases of human and/or agricultural importance. By tackling the causal factors of climate change, including air pollution, we holistically work to mitigate the risk of vector-borne diseases [33].

Electrifying vehicles offers an opportunity to reduce the amount of air pollution generated by traffic-related emissions. A review of available evidence has found that electric vehicles (EV) reduce air pollution, noting that the scale of these benefits depends on factors such as the type of EV and the source of energy generation [34]. In California, the benefits to health from the current rate of EV transition are already being seen. It has been estimated that an increase in EVs in a postcode (20 EVs per 1,000 people) is associated with a 3.2% reduction in asthma presentations in the emergency department [35].

EVs may already be leading to improvements in some measures; however, they are not without risk. A report into vehicle fires has shown that emissions of gaseous pollutants are higher when battery-powered electrical vehicles are involved, compared with internal combustion vehicles [36]. Emissions of hydrogen fluoride were many times greater in the EV fires [36]. Metal emissions were far higher in the electrical vehicle fires for all metals tested, but especially for nickel, cobalt, lithium, and manganese [36]. Non-exhaust emissions like harmful particles and particulate metals from tires are a serious issue unregulated yet, despite the type of EV or not. EV fires can start through spontaneous ignition, charging errors, and high-speed collisions, and once started, they are difficult to stop [37]. It is not just EVs at risk of these fires but the batteries themselves [36]. Figures from Australia show that structural fires caused by lithium-ion batteries igniting have almost doubled, and those fires escalate quickly and are difficult to extinguish [38].

Understanding these risks can be helpful during the transition to electric vehicles. The authors of the RISE report conclude by suggesting this information can be used in risk assessment and guidelines to strengthen the safety of electric vehicles [36]. The potential risks highlight the need for a thoughtful transition to new technologies to ensure they do not introduce new air pollution hazards. In fact, a focus on electrifying vehicle fleets may not be the best option when it comes to improving air quality and human health.

An assessment of a proposed cap and invest programme in New York City estimated the gains to children’s health under three emission reduction strategies: a CO_2_ cap imposed on fuel suppliers combined with investment into either 1) clean fuels and electrifying vehicles, 2) investment into public transport and active transport networks, or 3) a hybrid model [39]. The most effective model was one with a 25% reduction in CO_2_ and investment in public transport. Under this model, reductions in PM_2.5_ and NO_2_ were associated with significant reductions in cases of respiratory conditions in children and preterm births [39]. Using a conservative estimate, this equated to a USD$22 million saving to the health system [39]. The Lancet Commission on Pollution and Health estimated that for every dollar invested in the control of ambient air pollution in the USA, around USD$30 would be returned in benefits [40]. Taken together, these data show the benefits of investment in reducing air pollution, which led to both health and monetary savings. It is incumbent on us in public health to ensure that the technologies and investments in combatting climate change are carefully considered for their risk and benefits and are equitable.

The impact of climate change on human health can be seen through the vulnerability of populations, their level of exposure, and the resulting health outcomes [22]. These factors clearly demonstrate the need to urgently reduce greenhouse gas emissions. The elderly population, aged 65 years and older, is especially susceptible to the health impacts of climate change, particularly from extreme heat events [22]. However, children around the world are among the most severely impacted by the effects of climate change [22,23]. Prolonged exposure to air pollution can lead to asthma, respiratory problems, mental disorders, and complications during pregnancy that may result in infant mortality or long-term health issues in adulthood.

## Air pollution and children’s health

Children are among the most vulnerable to the adverse effects of air pollution exposure due to a combination of their immature physiology and metabolic pathways, as well as their hygiene and behaviours [41]. Many studies have demonstrated the impacts of air pollution on children’s health [42,43,44,45,46,47]. Exposures occurring early in life can damage developing cells and tissues, and that damage can carry on to health effects later in life [41]. Adverse effects start during the prenatal period, with particulate matter shown to be present on the foetal side of the placenta. Maternal exposure to air pollution is associated with adverse birth outcomes and pregnancy complications [48,49,50], and both maternal and child exposure to fine and ultrafine particulate matter can impact the development of children’s respiratory system, immune status, brain development, and cardiometabolic health [51]. While there is robust epidemiological evidence, the biological mechanisms underlying most risks associated with air pollution are not fully comprehended [52]. There is evidence that several criteria air pollutants affect the methylation of genes involved in immune modulation and impact immune cells, as well as increasing the risk of respiratory tract infections [53].

Children are facing some critical issues of public health relevance, and several of these overlap with air pollution. As an example:

- The 2022 Global Asthma Report found that 10% of children have asthma, with up to half of these children experiencing severe symptoms that interfere with their daily lives [54]. It has been shown that particulate matter from wildfire smoke can trigger asthma attacks in children more than other sources of particulate matter [55]. The new contaminants of concern, liquid crystal monomers, have been shown to be inhaled on fine particles and induce oxidative stress in lung cell models [19,56].
- Childhood obesity is of increasing concern, with the American Academy of Paediatrics releasing clinical practice guidelines in 2023 suggesting the issue is pressing enough that obese children over the age of 12 should be considered for weight-loss drugs. While obesity is complex and multifactorial, there is emerging evidence that exposure to higher particulate matter is also associated with higher odds of being overweight or obese [45].
- Air pollution exposure has been linked to poorer executive functioning and a reduction in academic achievement due to the effects on brain development and mental health [44,55]. This is particularly concerning as we emerge from the COVID-19 pandemic. UNICEF estimated in early 2022 that around 147 million children had missed more than half of their in-person schooling due to lockdowns [57]. In response, children were lagging in their foundational literacy and numeracy.
- A variety of social changes have led to a reduction in time spent outdoors for children, with this trend heightened during the COVID-19 pandemic [58]. As discussed earlier, higher levels of pollutants indoors mean that children are potentially exposed to air pollution at higher levels as they spend more time each day in these indoor environments. This makes it more pressing to quantify exposure to air pollutants indoors and find solutions.

The COVID-19 pandemic did highlight an opportunity for improving air quality in schools. COVID-19 forced a focus on improved ventilation, which is beneficial to indoor air quality as well as reducing exposure to infectious diseases [59]. We should advocate for schools around the world to continue with ventilation improvements as part of their strategy for a safe school environment. After home, children spend most of their day at school, so reducing exposure in the classroom can lead to a significant decrease in daily air pollution exposure. Another opportunity is the improved affordability and accessibility of low-cost air quality sensors. These small and easy-to-use sensors make it possible for families to explore their indoor air quality and reduce pollutant-generating activities, albeit limited to major pollutants such as particulate matter.

Given their unique vulnerability, children’s health should be at the forefront of our criteria when assessing strategies to reduce air pollution exposure in homes, schools, and ambient air.

## Future trends and prediction of air pollution concentration and its health impacts

As highlighted by the Lancet Commission on Pollution and Health and the World Health Organization (WHO), air pollution should be among the primary concerns due to the fact that it alone results in more than 6 million premature deaths each year. Besides children who are affected prenatally and postnatally, older people and those with respiratory conditions are at higher risk of air pollution-related health effects. Thus, it is important to understand the future trends and predictions of air pollution concentration and its health impact so that proper mitigation measures can be further taken. It has been identified that most residents in 171 countries in the world are experiencing various pollutant levels exceeding the international health guidelines, indicating the risks of health outcomes for many vulnerable groups of people [60]. This problem might get worse due to climate change, which has been known to be associated with changes in pollutant levels in the environment. Various pollutants in the environment may degrade the air quality, and long-term exposure to these pollutants may undergo alterations over time. These changes could potentially result in changes in the incidence of various health effects, including respiratory symptoms in children, as well as long-term outcomes that manifest as the children transition into adulthood [55]. New evidence has also been documented on the adverse health effects of air pollution at lower exposure levels, indicating the challenges in the areas [61].

The global trend in air pollution has shown a decrease in high-income countries due to regulations, yet it remains a significant concern in LMICs [61]. Anticipated in 2050, there is an expected rise in premature deaths attributed to exposure to particulate matter and ground-level ozone, with the highest likelihood occurring in China and India [62]. Thus, future research is essential in these countries, particularly in areas with elevated exposures but limited data.

Air pollution research faces challenges due to shifting pollution sources and characteristics [61]. Unhealthy air quality days vary every year and are impacted by pollution emissions as well as by natural events such as dust storms and wildfires and variations in weather [63]. Ongoing research is necessary to tackle this multifaceted issue. Table 1 outlines potential areas for future investigations focusing on indoor air quality, the intersection between climate change and air pollution, as well as air pollution’s and children’s health.

**Table 1:** Recommendations and opportunities for future studies in the field of air quality.


AREA	RECOMMENDATIONS AND OPPORTUNITIES

**Indoor air pollution**	Further research on the factors influencing IAQ and their impacts on health.

	Promote natural ventilation in building design to improve IAQ with focus on green building.

	Address the lack of access to clean fuels for cooking and heating in low-income and some regions of high-income countries.

	Implement government policies to phase out the use of dirty solid fuels for energy sources in homes, especially in LMICs.

	Research on the development and implementation of policies to address air pollution resulting from changing energy structures in urbanising regions and their effectiveness in mitigating health and environmental impacts.

	Strengthen controls and regulations for indoor smoking and e-cigarettes to reduce second- and third-hand smoke exposure.

	Investigate and address non-combustion sources and emerging contaminants, such as liquid crystal monomers, through systematic control strategies and further research.

**Climate change and air pollution**	Explore how projected changes in pollutants will affect current strategies to ensure pollutant remains below regulatory limits and whether new strategies are required, especially for ozone and particulate matter.

	Increase public awareness regarding the health risks associated with air pollution and climate change through strategies such as web apps, which allow targeted and individual recommendations.

	Formulate policies to mitigate both air pollution and climate change, recognizing their interconnectedness and potential health consequences.

	Encourage the adoption of EV to reduce traffic-related air pollution while addressing associated risks and ensuring safety measures.

	Invest in public transport for emission reduction goals with broader health benefits.

	Conduct thorough cost-benefit analyses of proposed emission reduction strategies, considering both health and monetary savings.

	Enhance health education efforts, particularly targeting vulnerable populations, to address the health impact of prolonged exposure to air pollution and climate change.

**Air pollution and children’s health**	Research is needed to comprehensively understand the biological effects and genetic risk associated with air pollution.

	More research is needed into improving home environments due to children’s reduced time spent outdoors and the potential for higher pollutant levels indoors.

	Strengthen regulations around smoking near children to safeguard their health and well-being.

	Consider children as central in climate change response, focusing on vulnerabilities, adaptive strategies, and policy for more inclusive and sustainable future.

	Research to investigate the link between air pollution and the exacerbation of asthma in children, looking at different sources of particulate matter to provide valuable insights of asthma management and prevention.

	More research on intersection of major health issue in children and air pollution.

	Research and advocacy for continued ventilation improvements in schools for safe school environment.


## Summary

Currently, we face new challenges to our air quality as well as new opportunities to find solutions. The evidence is already emerging that climate change is impacting air quality. Meteorological pattern changes in the last 60 years have been linked to a climate penalty for ozone in the Colorado Front Range. This climate penalty was seen in virtually all regions of the study area and accounted for an increase in ozone of between 0.5 ppb and 1 ppb [21]. This climate penalty means strategies to reduce air pollution may be less effective or take longer to achieve than expected [21]. Changing weather patterns, such as increased humidity and more sunny days, are expected to increase air pollutants, particularly ground-level ozone [46]. Wildfires, a significant contributor to annual particle air pollution, are increasing globally, with 152 countries experiencing an increase in exposure between 2015–2018 (compared with 2001–2004) [14]. The Organisation for Economic Co-operation and Development projected the risks from inaction and found that by 2050, particulate matter and ozone will become the top causes of environmentally-related deaths [62]. Further, they predict that PM_10_ in 2050 will be higher than in 2010 in South Asia, India, Indonesia, and Africa. In every region, they predict that significantly more premature deaths will be caused by ground-level ozone than in 2010. These are concerning predictions.

In the face of these challenges, opportunities to improve air quality are occurring. The transition to renewables and electric vehicles is underway and will offer reductions in air pollutants related to ill health as well as greenhouse gases [35]. The COVID-19 pandemic led to global lockdowns and changes in activity patterns that led to temporary, but dramatic, improvements in air quality and the built environment. Every region of the world experienced improved ambient air quality during this period, with dramatic reductions seen for NO_2_ (20–80%), NO_X_ (20–70%), black carbon (20–65%) and PM_2.5_ (10–40%) [47]. Water pollution also experienced a dramatic decrease during the pandemic, with an increase in river health and coastal environment quality [64]. While large-scale reductions in human activity are the exception, the example of COVID-19, combined with the significant gains seen by countries undertaking decisive action on environmental pollution [40], shows us that government policy and technological advances offer us a path forward to improving global air quality and the overall environment.

We are in a time of both opportunities and challenges to achieving clean air for all. The WHO recently issued updated guideline targets for several air pollutants, which take into account the body of evidence showing harm is associated with exposure at levels previously thought to represent good air quality [65]. The WHO acknowledges that their guidelines still focus on single-pollutant exposure, whereas we are exposed to complex combinations of air pollutants every day [65], Future work needs to focus on understanding which climate adaptation strategies offer co-benefits for clean air; the use of modern statistical methods and the emergence of low-cost sensors to gather more data on exposure to combined exposures of air pollutants, especially indoors; and identifying those populations most at risk from air pollution exposure and developing research-based treatment plans.
